# Genetic homocysteine risk shapes cardiocerebral structure and multimorbidity through age- and sex-specific mechanisms: a UK Biobank study

**DOI:** 10.3389/fnut.2025.1637592

**Published:** 2025-11-14

**Authors:** Chenjie Feng, Yupeng Ma, Tian Zhang, Peng Zhang, Yu Zhao

**Affiliations:** 1 College of Medical Information and Engineering, Ningxia Medical University, Yinchuan, China; 2 School of Public Health, Ningxia Medical University, Yinchuan, China; 3 Ningxia Key Laboratory of Environmental Factors and Chronic Disease Control, Yinchuan, China

**Keywords:** homocysteine, sulfur microbial diet, EAT-Lancet, cardiovascular structure, cardiocerebrovascular diseases, UK Biobank

## Abstract

**Background:**

Elevated homocysteine (Hcy) levels have been implicated in cardiometabolic and neurological disorders. However, the age- and sex-specific mechanisms by which genetically determined Hcy levels contribute to disease risk via structural alterations in the heart and brain remain unclear.

**Methods:**

We analyzed data from 306,796 UK Biobank participants. A weighted polygenic risk score (PRS) for Hcy was constructed and tested for associations with cardiovascular and neuroimaging phenotypes. Mediation analyses assessed the extent to which these structural traits mediated disease risk. We also examined whether two dietary patterns—the sulfur microbial diet and the EAT-Lancet diet—modulated Hcy levels or disease associations.

**Results:**

Genetically elevated Hcy was significantly associated with sex- and age- specific alterations in brain white matter and cardiac structure. These structural traits partially mediated the link between Hcy and hypertension, dyslipidemia, and cognitive impairment. Surprisingly, neither dietary index was associated with Hcy levels, although both showed independent associations with disease risk.

**Conclusion:**

Our findings suggest that genetically determined Hcy levels impact cardiocerebral structure in a sex- and age-dependent manner, contributing to disease risk. Structural imaging phenotypes offer potential as early mediators. The dietary effects on disease risk may involve pathways independent of Hcy modulation.

## 1 Introduction.

Multimorbidity, clinically defined as the co-occurrence of at least two chronic conditions within a single individual (1), has emerged as a critical global public health challenge exacerbated by demographic aging. Of these conditions, cardiocerebrovascular diseases (CVD) constitute the leading cause of morbidity and mortality (2).

While traditional risk factors such as hypertension and obesity are well-studied drivers of CVD (3, 4), emerging evidence highlights homocysteine (Hcy) as a potent yet underinvestigated mediator of cardiocerebral pathology (5). Hcy is a sulfur-containing, non-proteinogenic amino acid formed during the metabolism of methionine (6). Elevated circulating concentrations of Hcy, a condition termed hyperhomocysteinemia (HHcy), have been implicated in multiple age-related diseases, including neurovascular diseases (7), atherosclerosis (8), stroke (9, 10) and cognitive impairment. The pathogenic mechanisms underlying the deleterious effects of HHcy are complex and multifactorial, encompassing proposed pathways such as redox imbalance mediated by excessive reactive oxygen species production (11), dysregulation of nitric oxide (NO) signaling (12), induction of endothelial dysfunction driven by mitochondrial and endoplasmic reticulum stress (13), and potentiation of systemic inflammation (14). Notably, while these pathways predominantly implicate functional and metabolic derangements, no studies have systematically examined the structural consequences of Hcy on cardiovascular architecture, including ventricular remodeling, myocardial deformation, or alterations in tissue thickness, nor comprehensively evaluated its neuroanatomical effects. Addressing these gaps holds significant clinical relevance, as structural modifications often precede symptomatic manifestations by years and may serve as valuable preclinical biomarkers for timely therapeutic intervention (15). Consequently, elucidating Hcy-mediated structural damage in both cardiac and cerebral tissues represents a crucial missing component in understanding its pathophysiological role in multimorbidity, particularly in CVD diseases.

Circulating Hcy levels represent a complex phenotype co-regulated by polygenic inheritance and environmental modulation. Genetic architecture accounts for the predominant share of phenotypic variance, with heritability estimates attaining 66% (16). This substantial genetic component enables GWAS to identify variants to function as robust instrumental variables for both predicting biomarker levels and investigating causal relationships with diverse phenotypic outcomes. However, the non-modifiable nature of genetic predisposition necessitates alternative intervention targets. In that cases, dietary modification presents itself as a clinically actionable environmental modulator of Hcy metabolism, providing viable therapeutic avenues for Hcy homeostasis restoration and primary prevention of Hcy-associated morbidities. Beyond well-established dietary models such as the Mediterranean (17) and Dietary Approaches to Stop Hypertension (DASH) diets (18), emerging patterns like the sulfur microbial diet (19) and the EAT-Lancet diet (20) are garnering attention for their biological effects. The sulfur microbial diet, as measured by the Sulfur Microbial Diet Score (SMDS), has been associated with an increased susceptibility to colorectal cancer (21, 22), obesity (23), and non-alcoholic fatty liver disease (24). This dietary pattern represents a pro-inflammatory and potentially pathogenic nutritional profile. We hypothesize that the sulfur microbial diet may influence Hcy levels through direct perturbation of sulfur-dependent Hcy metabolism (25) and indirect modulation of gut microbial communities involved in sulfur cycling, which could affect Hcy clearance. In contrast, the predominantly plant-based EAT-Lancet diet has been linked to multi-system benefits, with randomized trials and observational studies associating this plant-forward regimen with enhanced cognitive performance, improved cardiometabolic parameters (26), and reduced diabetes incidence (27). This nutritionally optimized, sustainable dietary approach exemplifies a health-promoting eating pattern. The rich profile of plant-derived nutrients such as folate, vitamin B6, and vitamin B12 may attenuate circulating Hcy concentrations through improved biological availability of these essential enzymatic cofactors in the Hcy metabolic pathway (28). However, no study has systematically investigated the relationships between these two diet patterns and Hcy, nor quantified their relative contributions to disease pathogenesis. Considering the substantial intervenability of dietary patterns, the mechanistic investigation of Hcy homeostasis modulation by specific dietary patterns is crucial for advancing nutraceutical approaches to CVD prevention through precision nutrition paradigms.

In this regard, phenome-wide association study (PheWAS) (29) design, leveraging comprehensive phenotypic data from large-scale biobanks like UK Biobank encompassing cardiovascular functional parameters, neuroimaging metrics, and dietary profiles, provides a unique opportunity to investigate associations between genetically predicted Hcy levels and diverse cardiocerebral structural phenotypes in middle-aged and older populations, while concurrently evaluating the modulatory effects of key intervenable dietary factors such as sulfur microbial diet and EAT-Lancet diet on Hcy concentrations. This integrative approach offers novel insights into potential early structural biomarkers of Hcy-related pathology and actionable dietary interventions for precision prevention.

## 2 Methods

### 2.1 Study population

Our analysis included 306,796 participants from the UK Biobank cohort, aged 37–73 years at recruitment. All participants underwent comprehensive baseline assessments comprising standardized questionnaires, physical measurements, and biospecimen collection. The study protocol received ethical approval from the North West Multicenter Research Ethics Committee, with written informed consent obtained from all participants prior to enrollment.

### 2.2 Sulfur microbial diet assessment

The sulfur microbial diet score was calculated from five administrations of the Oxford WebQ 24-h dietary recall (30), quantifying consumption of 43 sulfur-metabolizing bacteria-associated foods such as processed meat, liquor, and low-calorie drinks, beer, fruit juice, legumes, mixed vegetables, and sweets/desserts. Weighting coefficients for each dietary component were established based on a study by Nguyen et al. (21), incorporating both reduced rank regression and stepwise linear regression approaches. Higher scores indicate greater consumption of sulfur-metabolizing bacteria-related foods and consequently reflect higher predicted abundance of sulfur-metabolizing bacteria in the gut microbiota. Supplementary Table 1 presents the specific components of the sulfur microbial diet and their respective coefficients. Participants with incomplete or unreliable 24-h dietary recall data or missing covariate information were excluded from analysis. For outlier values, we implemented median imputation to maintain data integrity while minimizing bias.

### 2.3 The EAT-Lancet diet assessment

The EAT-Lancet diet assessment score was calculated based on the methodology described by Knuppel et al. (20), encompassing eight key dietary categories including whole grains, tubers and starchy vegetables, vegetables, fruits, dairy foods, protein sources, added fats, and added sugars. Participants received 1 point per met recommendation (0 if unmet), yielding a total score from 0 to 14. Detailed criteria for the assessment of these EAT-Lancet components are provided in Supplementary Table 2.

### 2.4 Weighted genetic risk scores of Hcy

Weighted genetic risk scores (wGRSs) for Hcy were derived from 14 independent genome-wide significant SNPs (*p* < 5 × 10^−8^, *r*^2^ < 0.01) identified in European-ancestry GWAS of 44,147 individuals (31). These variants collectively explained 3.22% of Hcy variance (Supplementary Table 3). The wGRSs were calculated by summing risk alleles weighted by their respective effect sizes from the discovery GWAS (31). All 14 SNPs satisfied the criterion for strong predictive power (*F*-statistics > 10), indicating robust instrument strength for subsequent analyses.

### 2.5 Outcome assessment

Outcome variables in this study were comprehensively assessed from the UKBB dataset, encompassing cardiovascular function parameters, neuroimaging-derived brain volumes, and CVD disorders.

Assessment of cardiovascular function and brain structure was based on measures derived from the UKBB imaging datasets. Cardiovascular parameters included key ventricular volumetric measures, specifically right ventricular end-systolic volume (RVESV) and left ventricular end-systolic volume (LVESV). Myocardial mechanics were characterized through the analysis of myocardial strain across multiple dimensions, including end-systolic circumferential strain (basal segment), left ventricular (LV) longitudinal strain (Segment 6), and LV radial strain. The LV radial strain was further partitioned according to the American Heart Association (AHA) 17-segment model, providing both global radial strain and regional strain for AHA segments 9, 10, and 15. Structural cardiac indices were represented by LV mean myocardial wall thickness (AHA segment 9). Neuroimaging metrics primarily focused on brain volumetric assessment, with white matter volume quantified from T1-weighted magnetic resonance imaging data.

CVD were ascertained through linkage to UKBB clinical records with data corresponding to field I65. This encompassed a broad spectrum of diagnoses, including vascular pathologies such as aortic aneurysm and dissection, arterial embolism and thrombosis, atherosclerosis, cerebral hemorrhage, cerebral infarction, occlusion of cerebral and pulmonary arteries, and subarachnoid hemorrhage. Cardiac conditions included ischemic heart disease (IHD) and other specified heart diseases. Metabolic disorders comprised diagnoses of diabetes, hypercholesterolemia, hypertension, obesity, and metabolic syndrome. Additionally, relevant sequelae, such as non-trauma intracerebral hemorrhage (NTIH) and other sequelae of cerebrovascular diseases, were included. The specific UKBB field IDs and corresponding ICD-10 codes used for defining these outcomes are fully documented in Supplementary Table 4.

### 2.6 Correlation analyses

Our analytical framework was designed to systematically investigate the complex interrelationships among dietary patterns, genetically predicted Hcy levels, and clinical indicators and CVD. A multi-stage regression approach was employed to dissect these associations.

Initially, linear regression models were utilized to quantify the associations between genetically predicted Hcy levels and key biological indicators, including measures of cardiovascular function and neuroimaging-derived brain volumes. Simultaneously, linear regression was also applied to assess the relationships between two distinct dietary models (the sulfur microbial diet and the EAT-Lancet diet) and Hcy levels. Subsequent Cox proportional hazards modeling evaluated the dual risk pathways including Hcy-dependent cerebrovascular cardiometabolic disorders through Hcy-incident event associations, and diet-mediated disease risk via direct dietary pattern-outcome relationships, thereby enabling discrimination between Hcy-mediated and Hcy-independent pathological mechanisms.

### 2.7 Statistical analyses

All statistical models were adjusted for potential confounders, including age, sex, and the first five principal components of population stratification (PC1-PC5). Given the established sexually dimorphic nature (32), we dual stratification analyses by age (< 65 vs. ≥65 years) and sex to systematically examine subgroup-specific associations between Hcy levels with heart-related indicators and brain region volumes, the relationships between Hcy levels and diseases, the dietary models on Hcy levels, and dietary models on diseases within age and sex subgroups.

The distribution of baseline characteristics was evaluated across quartiles of the sulfur microbial diet and the EAT-Lancet diet score. Continuous variables are presented as means ± standard deviations (SD), while categorical variables are expressed as percentages (%). WGRSs of Hcy levels were performed with PRSice-2 software (version 2.3.3).

## 3 Results

### 3.1 Baseline population characteristics

The study included 110,167 females (94,303 aged < 65; 15,864 aged ≥ 65) and 89,767 males (72,376 aged < 65; 17,391 aged ≥ 65) (Table 1). Significant age-related differences (p < 0.05) were observed in both sexes for most variables. Older participants (≥65 years) had higher mean BMI in females (26.8 vs. 26.5, *p* < 0.001) but slightly lower BMI in males (27.4 vs. 27.5, *p* = 0.006). Cardiometabolic diseases were more prevalent in older adults.

**Table 1:** Baseline demographic and clinical characteristics of the study population stratified by sex and age group (*N* = 199,934).

**Variables**	**Levels**	**Female (N** = **110,167)**			**Male (N** = **89,767)**		
		<**65 (*****N** =* **94,303)**	>**65 (*****N** =* **15,864)**	**pval**	<**65 (*****N** =* **72,376)**	>**65 (*****N** =* **17,391)**	**pval**
BMI	Mean ± SD	26.5 ± 5.1	26.8 ± 4.6	**< 0.001**	27.5 ± 4.2	27.4 ± 3.8	**0.006**
PC1	Mean ± SD	−2.7 ± 49.1	−7.8 ± 31.5	**< 0.001**	−3.6 ± 46.4	−8.4 ± 26.9	**< 0.001**
PC2	Mean ± SD	0.5 ± 27.4	1.7 ± 19.3	**< 0.001**	0.7 ± 24.5	1.6 ± 18.2	**< 0.001**
PC3	Mean ± SD	−0.7 ± 14.1	−0.7 ± 10.4	0.665	0.1 ± 13.5	−0.2 ± 10.7	**0.002**
PC4	Mean ± SD	−0.5 ± 10.7	−0.6 ± 10.9	0.071	−0.4 ± 10.9	−0.3 ± 10.5	0.853
PC5	Mean ± SD	−0.4 ± 7.3	−1.0 ± 7.2	**< 0.001**	−0.4 ± 7.2	−1.1 ± 7.1	**< 0.001**
Ethnic	Prefer not to answer	185 (0.2%)	36 (0.2%)	**< 0.001**	260 (0.4%)	93 (0.5%)	**< 0.001**
	Do not know	27 (0%)	4 (0%)		25 (0%)	3 (0%)	
	White	58 (0.1%)	8 (0.1%)		73 (0.1%)	13 (0.1%)	
	Mixed	8 (0%)	0 (0%)		3 (0%)	1 (0%)	
	Asian or Asian British	7 (0%)	0 (0%)		2 (0%)	0 (0%)	
	Black or Black British	3 (0%)	0 (0%)		1 (0%)	0 (0%)	
	Chinese	346 (0.4%)	26 (0.2%)		178 (0.2%)	22 (0.1%)	
	Other ethnic group	768 (0.8%)	77 (0.5%)		526 (0.7%)	47 (0.3%)	
	British	83,282 (88.3%)	14,462 (91.2%)		64,526 (89.2%)	16,140 (92.8%)	
	Irish	2,215 (2.3%)	379 (2.4%)		1,867 (2.6%)	376 (2.2%)	
	Any other white background	4,318 (4.6%)	630 (4%)		2,440 (3.4%)	398 (2.3%)	
	White and Black Caribbean	143 (0.2%)	8 (0.1%)		68 (0.1%)	7 (0%)	
	White and Black African	92 (0.1%)	7 (0%)		41 (0.1%)	0 (0%)	
	White and Asian	202 (0.2%)	22 (0.1%)		154 (0.2%)	9 (0.1%)	
	Any other mixed background	248 (0.3%)	11 (0.1%)		116 (0.2%)	21 (0.1%)	
	Indian	774 (0.8%)	80 (0.5%)		809 (1.1%)	132 (0.8%)	
	Pakistani	97 (0.1%)	10 (0.1%)		177 (0.2%)	20 (0.1%)	
	Bangladeshi	17 (0%)	0 (0%)		15 (0%)	2 (0%)	
	Any other Asian background	237 (0.3%)	27 (0.2%)		220 (0.3%)	42 (0.2%)	
	Caribbean	845 (0.9%)	48 (0.3%)		409 (0.6%)	36 (0.2%)	
	African	373 (0.4%)	21 (0.1%)		432 (0.6%)	23 (0.1%)	
	Any other Black background	22 (0%)	4 (0%)		8 (0%)	2 (0%)	
Smoking	Mean ± SD	0.5 ± 0.6	0.5 ± 0.6	0.487	0.6 ± 0.7	0.6 ± 0.6	**< 0.001**
Alcohol	Mean ± SD	1.9 ± 0.4	1.8 ± 0.5	**< 0.001**	1.9 ± 0.3	1.9 ± 0.3	0.154
Total energy	Mean ± SD	8,244.8 ± 2,863.5	8,148.5 ± 2,794.6	0.016	9,814.9 ± 3,506.3	9,428.9 ± 3,072.8	**< 0.001**
Education	Mean ± SD	0.8 ± 0.4	0.7 ± 0.5	**< 0.001**	0.8 ± 0.4	0.7 ± 0.4	**< 0.001**
Aortic aneurysm and dissection	0	94,086 (99.8%)	15,753 (99.3%)	**< 0.001**	71,617 (99%)	16,870 (97%)	**< 0.001**
	1	217 (0.2%)	111 (0.7%)		759 (1%)	521 (3%)	
Arterial embolism and thrombosis	0	94,177 (99.9%)	15,821 (99.7%)	**< 0.001**	72,125 (99.7%)	17,227 (99.1%)	**< 0.001**
	1	126 (0.1%)	43 (0.3%)		251 (0.3%)	164 (0.9%)	
Atherosclerosis	0	94,046 (99.7%)	15,740 (99.2%)	**< 0.001**	71,914 (99.4%)	17,018 (97.9%)	**< 0.001**
	1	257 (0.3%)	124 (0.8%)		462 (0.6%)	373 (2.1%)	
Cerebral hemorrhage	0	94,083 (99.8%)	15,756 (99.3%)	**< 0.001**	72,124 (99.7%)	17,238 (99.1%)	**< 0.001**
	1	220 (0.2%)	108 (0.7%)		252 (0.3%)	153 (0.9%)	
Cerebral infarction	0	93,453 (99.1%)	15,387 (97%)	**< 0.001**	71,056 (98.2%)	16,595 (95.4%)	**< 0.001**
	1	850 (0.9%)	477 (3%)		1,320 (1.8%)	796 (4.6%)	
Diabetes	0	90,085 (95.5%)	14,536 (91.6%)	**< 0.001**	66,291 (91.6%)	14,879 (85.6%)	**< 0.001**
	1	4,218 (4.5%)	1,328 (8.4%)		6,085 (8.4%)	2,512 (14.4%)	
Hypercholesterolemia	0	86,003 (91.2%)	12,454 (78.5%)	**< 0.001**	60,624 (83.8%)	11,867 (68.2%)	**< 0.001**
	1	8,300 (8.8%)	3,410 (21.5%)		11,752 (16.2%)	5,524 (31.8%)	
Hypertension	0	75,306 (79.9%)	8,855 (55.8%)	**< 0.001**	51,484 (71.1%)	8,014 (46.1%)	**< 0.001**
	1	18,997 (20.1%)	7,009 (44.2%)		20,892 (28.9%)	9,377 (53.9%)	
IHD	0	89,757 (95.2%)	13,842 (87.3%)	**< 0.001**	63,371 (87.6%)	12,848 (73.9%)	**< 0.001**
	1	4,546 (4.8%)	2,022 (12.7%)		9,005 (12.4%)	4,543 (26.1%)	
Metabolic disorders	0	82,547 (87.5%)	11,327 (71.4%)	**< 0.001**	58,151 (80.3%)	10,687 (61.5%)	**< 0.001**
	1	11,756 (12.5%)	4,537 (28.6%)		14,225 (19.7%)	6,704 (38.5%)	
NTIH	0	94,224 (99.9%)	15,827 (99.8%)	**< 0.001**	72,208 (99.8%)	17,290 (99.4%)	**< 0.001**
	1	79 (0.1%)	37 (0.2%)		168 (0.2%)	101 (0.6%)	
Obesity	0	88,266 (93.6%)	14,663 (92.4%)	**< 0.001**	67,554 (93.3%)	16,050 (92.3%)	**< 0.001**
	1	6,037 (6.4%)	1,201 (7.6%)		4,822 (6.7%)	1,341 (7.7%)	
Occlusion	0	94,145 (99.8%)	15,732 (99.2%)	**< 0.001**	71,998 (99.5%)	17,109 (98.4%)	**< 0.001**
	1	158 (0.2%)	132 (0.8%)		378 (0.5%)	282 (1.6%)	
Other cerebral blood vessel	0	93,313 (99%)	15,245 (96.1%)	**< 0.001**	71,333 (98.6%)	16,552 (95.2%)	**< 0.001**
	1	990 (1%)	619 (3.9%)		1,043 (1.4%)	839 (4.8%)	
Other heart diseases	0	87,370 (92.6%)	12,636 (79.7%)	**< 0.001**	62,473 (86.3%)	11,836 (68.1%)	**< 0.001**
	1	6,933 (7.4%)	3,228 (20.3%)		9,903 (13.7%)	5,555 (31.9%)	
Sequelae of cerebrovascular diseases	0	94,041 (99.7%)	15,731 (99.2%)	**< 0.001**	72,014 (99.5%)	17,195 (98.9%)	**< 0.001**
	1	262 (0.3%)	133 (0.8%)		362 (0.5%)	196 (1.1%)	
Subarachnoid_hemorrhage	0	94,025 (99.7%)	15,781 (99.5%)	**< 0.001**	72,207 (99.8%)	17,343 (99.7%)	0.348
	1	278 (0.3%)	83 (0.5%)		169 (0.2%)	48 (0.3%)	

“1” indicates the presence of the disease, and “0” indicates the absence of the disease. Statistical significance: p-values (pval) < 0.05 are bolded to indicate significant differences between age groups (< 65 vs. ≥65) within each sex.

BMI, Body Mass Index; PC1–PC5, Principal Components (derived from genetic or phenotypic data); IHD, Ischemic Heart Disease; NTIH, Non-Traumatic Intracranial Hemorrhage.

### 3.2 Sex-stratified homocysteine associations with cardio-cerebral structures across age

**Figure 1:**
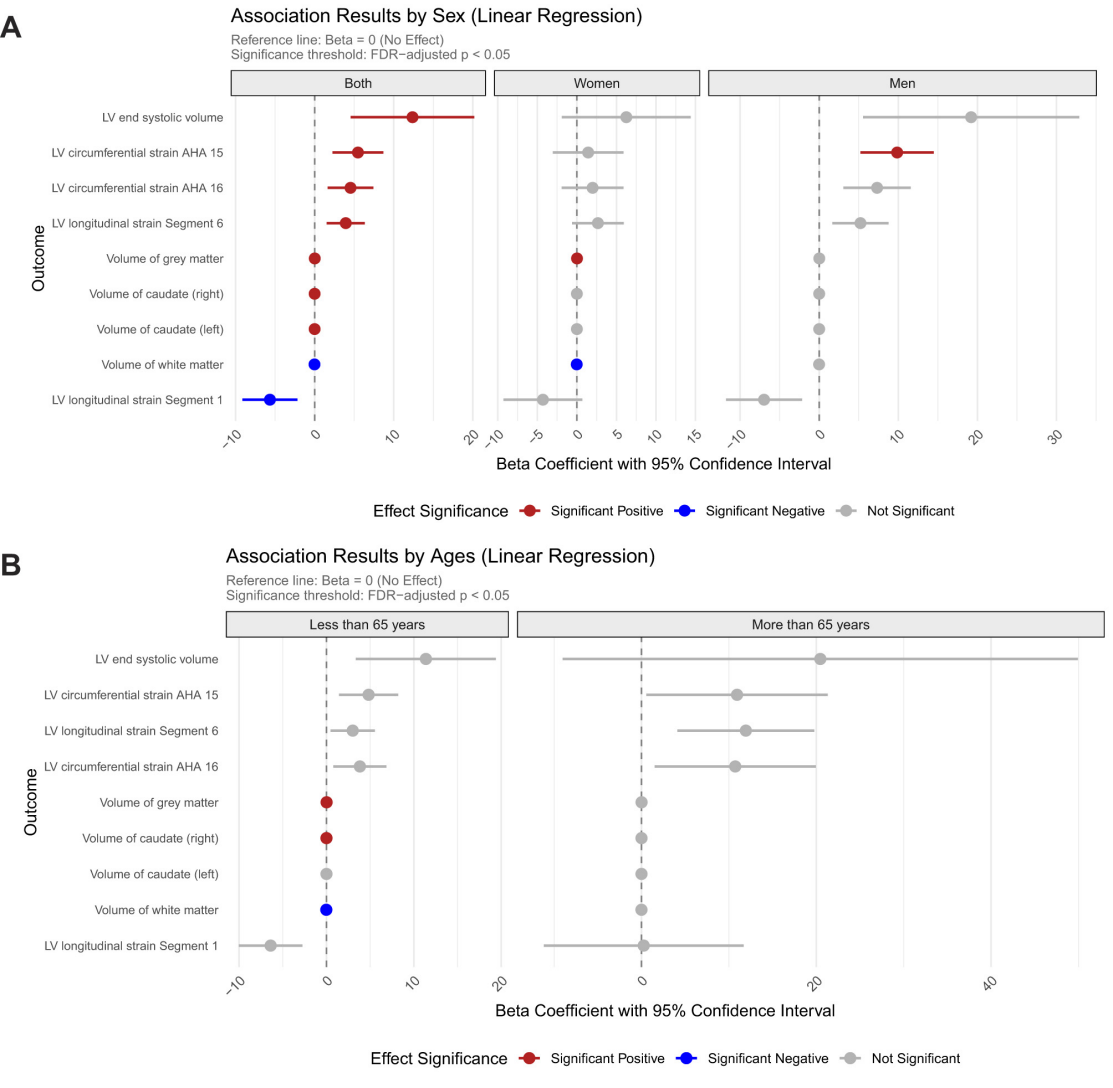
Sex- and age-specific associations between homocysteine levels and cardiovascular/brain structural parameters. **(A)** Association results stratified by sex; **(B)** Association results stratified by age group.

Our regression analyses demonstrated significant sex-stratified associations between Hcy levels and cardiocerebral structural phenotypes, with distinct patterns emerging in cardiovascular assessments. Across the entire cohort, higher Hcy levels were significantly linked to increased LVESV (β = 12.37, FDR_p = 0.047) (Figure 1A). Notably, male participants showed particularly strong associations in cardiac function metrics, with elevated Hcy levels correlating with impaired LV circumferential strain in the AHA 15 segment (β = 9.85, FDR_p = 0.005). Further segmental analysis uncovered complex regional relationships, where Hcy levels were positively associated with longitudinal strain in segment 6 (β = 3.93, FDR_p = 0.046), while showing an inverse relationship in segment 1 (β =−5.65, FDR_p = 0.046).

The cerebrovascular analyses revealed region-specific associations. Bilateral caudate nuclei volumes displayed nominally positive associations with Hcy levels (left: β = 2.1 × 10^−3^; right: β = 2.4 × 10^−3^), and gray matter volume showed a transient positive association in the overall cohort (β = 1.5 × 10^−2^, FDR_p = 1.5 × 10^−3^) that disappeared upon sex stratification. Notably, white matter volume demonstrated significant negative associations with Hcy levels in both the full cohort (β =−1.6 × 10^−2^, FDR_p = 3.4 × 10^−4^) and the female subgroup (β =−1.8 × 10^−2^, FDR_p = 1.2 × 10^−2^), with no observable effects in males (Figure 1A).

Age-stratified analyses uncovered modified vulnerability profiles, with younger participants under 65 demonstrating nominal positive associations for gray matter (β =1.6 × 10^−2^, FDR_p = 7.8 × 10^−4^) and volume of right caudate (β = 2.4 × 10^−4^, FDR_p = 3.5 × 10^−2^) but negative association with white matter volumes (β = −1.8 × 10^−2^, FDR_p = 7.7 × 10^−5^). Notably, these structural associations were completely attenuated in individuals aged ≥ 65 years, suggesting age-dependent susceptibility to Hcy-mediated cardiocerebral remodeling (Figure 1B).

### 3.3 Age-divergent Hcy risk profiles

**Figure 2:**
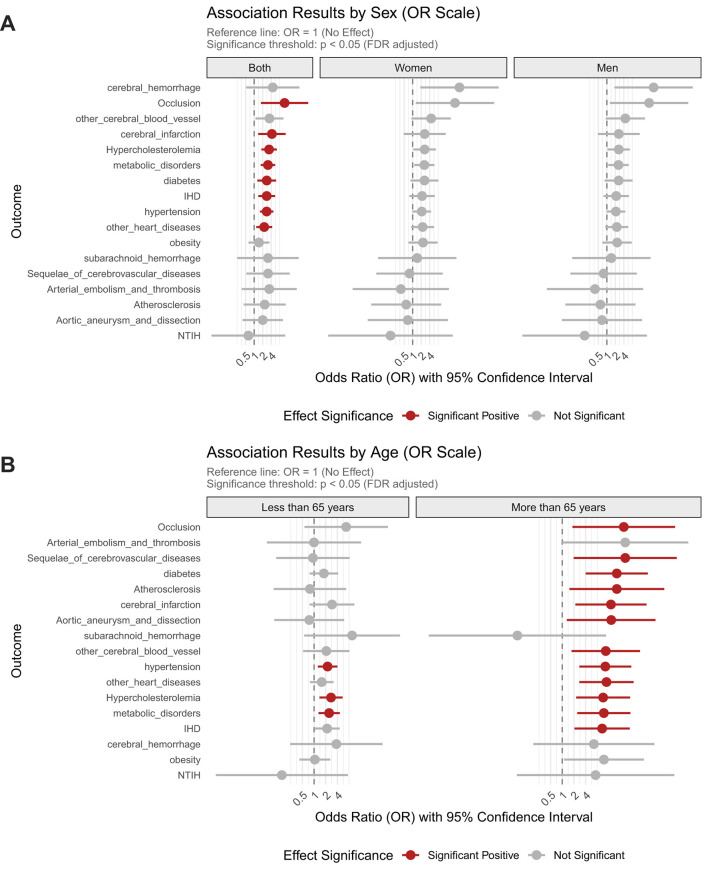
Sex- and age-stratified associations between homocysteine levels and the risk of cardiocerebrovascular and metabolic disorders. **(A)** Associations stratified by sex; **(B)** Associations stratified by age groups.

Genetically predicted Hcy as a pan-risk factor for diverse cardiocerebrovascular disorders, substantially increasing the risk of precerebral artery occlusion by 11.91-fold (FDR_p = 2.68 × 10^−2^), cerebral infarction by 4.24-fold (FDR_p = 2.68 × 10^−2^), hypercholesterolemia by 3.37-fold (FDR_p = 1.4 × 10^−3^), hypertension by 2.77-fold (FDR_p = 1.4 × 10^−3^), ischemic heart disease by 2.78 fold (FDR_p = 1.4 × 10^−2^), diabetes by 2.79-fold (FDR_p = 2.7 × 10^−2^). No gender differences were observed in these associations (Figure 2A).

The impact of elevated Hcy was particularly pronounced in older adults, where it dramatically amplified disease risk across CVD and metabolic conditions. Hcy increased the risk of precerebral artery occlusion by 38.6-fold, cerebral infarction by 18-fold, and cerebrovascular sequelae by 42-fold, while also elevating the likelihood of aortic aneurysm/dissection by 18.3-fold and ischemic heart disease by 10.7-fold. Metabolic disorders were similarly affected, with Hcy increasing diabetes risk 25.5-fold, hypercholesterolemia 11.3-fold, atherosclerosis 25.5-fold, and hypertension risk 12.9-fold in this age group. In contrast, younger individuals ( ≤ 65 years) exhibited markedly weaker associations, with Hcy elevating hypertension risk 2.2-fold, hypercholesterolemia 2.7-fold, and other metabolic disorders 2.4-fold, representing a 42-68% reduction in effect magnitude compared to elderly counterparts (Figure 2B).

### 3.4 Cardiocerebral mediation of Hcy-metabolic disease associations

In participants under 65, genetically elevated Hcy levels influenced metabolic disorders through distinct cardiocerebral pathways. For hypercholesterolemia, higher Hcy levels were linked to reduced gray matter volume and impaired left ventricular longitudinal strain in segment 1, both of which contributed to increased disease risk (Supplementary Figure 1). Similarly, diminished white matter volume (β = −0.13) and worsened LV segment 1 strain (β = −0.047) partially mediated the association with Hcy (Supplementary Figure 1). Notably, LV segment 1 strain served as a convergent mediator for both conditions, while neuroanatomical mediators exhibited disease specificity—gray matter predominated in lipid dysregulation whereas white matter dominated blood pressure modulation.

### 3.5 Age-modulated dietary impacts on cardiometabolic risk

**Figure 3:**
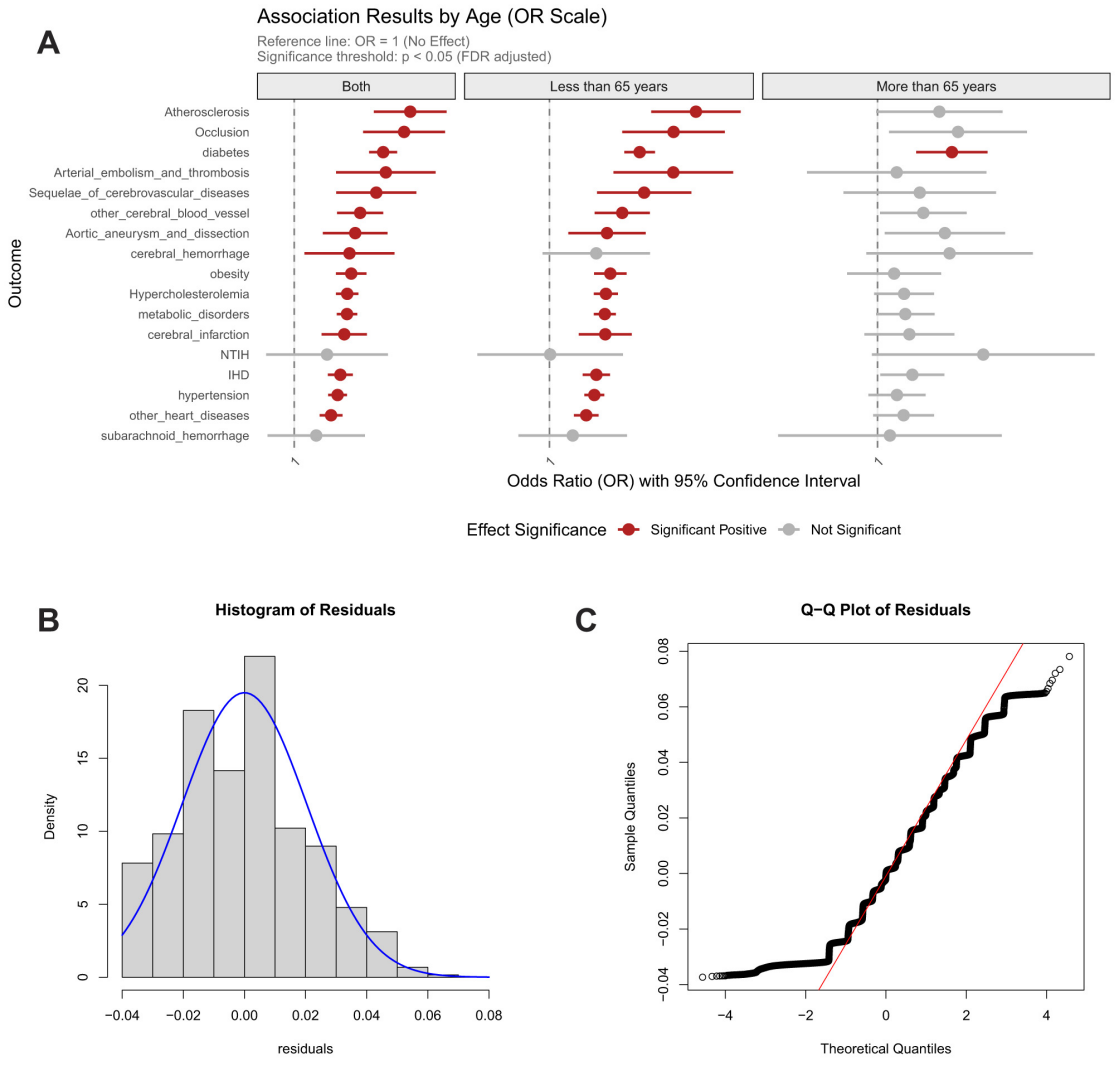
Association between sulfur microbial diet score and risk of cardiocerebrovascular diseases, with age-stratified analysis and model validation. **(A)** Age-stratified associations with cardiocerebrovascular diseases; **(B)** Residual distribution of the dietary model incorporating homocysteine weighted genetic risk score; **(C)** Quantile-quantile (Q–Q) plot for model evaluation.

Dietary pattern analyses uncovered age-dependent cardiometabolic risk profiles associated with distinct nutritional interventions. The sulfur-microbiota-associated diet increased diabetes risk by 1.09-fold consistently across all ages, while demonstrating particularly strong effects in older adults (combined: FDR_p = 2.23 × 10^−34^; < 65y: OR = 1.09; > 65y: OR = 1.07). In the population aged under 65 years, this dietary pattern elevated risk for multiple vascular and metabolic conditions, including atherosclerosis, arterial embolism/thrombosis, aortic aneurysm/dissection, post-stroke sequelae, cerebral infarction, other cerebrovascular diseases, cerebral hemorrhage, obesity, hypercholesterolemia, other metabolic disorders, ischemic heart disease, hypertension, other cardiac conditions (Figure 3).

**Figure 4:**
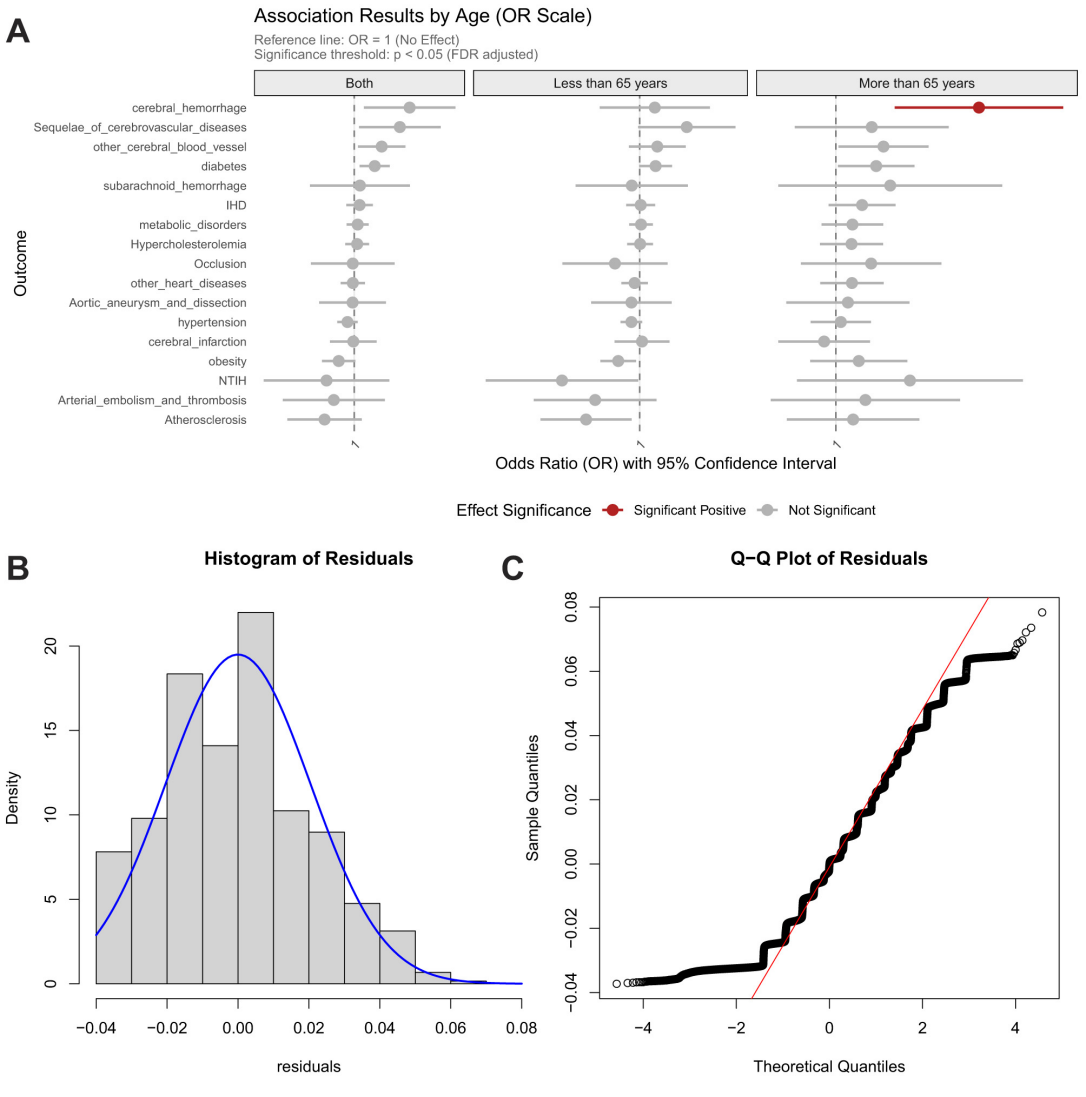
Association between EAT-Lancet diet score and risk of cardiocerebrovascular diseases, with age-stratified analysis and model validation. **(A)** Age-stratified associations with cardiocerebrovascular diseases; **(B)** Residual distribution of the dietary model incorporating homocysteine weighted genetic risk score; **(C)** Quantile-quantile (Q–Q) plot for model evaluation.

Contrastingly, the EAT-Lancet diet manifested selective risk potentiation, elevating hypertension risk specifically in the elderly population (>65 y) (Figure 4). Crucially, both dietary patterns showed null associations with genetically predicted Hcy levels, indicating their mechanistic dissociation from Hcy-mediated pathways. These findings collectively suggest that dietary influences on cardiometabolic health are predominantly age-stratified and operate through biological pathways independent of homocysteine regulation.

### 3.6 Sex-stratified dietary impacts on cardiometabolic risk

The sex-stratified analyses of dietary patterns on CVD revealed divergent cardiovascular and cerebrovascular risk profiles associated with the two dietary patterns (Supplementary Figures 2A, B). For the sulfur microbial diet, both women and men exhibited similar positive associations (FDR_p < 0.05) with multiple outcomes, including atherosclerosis, occlusion, arterial embolism and thrombosis, sequelae of cerebrovascular diseases, aortic aneurysm and dissection, obesity, hypercholesterolemia, metabolic disorders, cerebral infarction, IHD, hypertension, and other heart diseases (Supplementary Figure 2A). Conversely, the EAT-Lancet diet demonstrated no significant associations with any cardiometabolic or cerebrovascular outcome in either women or men (all FDR_p ≥ 0.05; Supplementary Figure 2B). Consistent with age-stratified results, sex-stratified analyses also showed no significant associations between either dietary pattern and genetically predicted Hcy levels.

## 4 Discussion

In the present analyses, we systematically investigated the associations between genetically predicted Hcy levels and cardiocerebral structural phenotypes and related diseases. Sex-stratified analyses revealed differential patterns that males exhibited stronger associations with cardiac metrics, whereas females demonstrated specificity for cerebrovascular phenotypes. Age stratification further highlighted enhanced Hcy-associated risks in individuals older than 65 years. Our findings also suggested that the effects of hypertension and hypercholesterolemia were potentially indirectly mediated by cardiocerebral metrics. Additionally, we examined the association between adherence to the sulfur microbial diet and the EAT-Lancet diet with genetically predicted Hcy levels, finding no significant association for either dietary pattern. However, considering that both dietary patterns are independently associated with CVD, this lack of association with Hcy suggests that their effects on cardiovascular health are likely mediated through mechanisms independent of homocysteine metabolism.

Our findings demonstrate significant associations between genetically predicted Hcy levels and cardiocerebral indicators, revealing notable sex-specific patterns. For cardiovascular phenotypes, our study demonstrated a significant association between genetically predicted Hcy levels and LVESV in the overall cohort and in males, which aligns with prior evidence that elevated Hcy can contribute to both systolic and diastolic left ventricular dysfunction, potentially progressing to chronic heart failure (33). Potential underlying mechanisms for these cardiovascular effects include oxidative stress, such as ROS overproduction (34), and pro-inflammatory cytokine activation (35). Notably, these cardiovascular associations were predominantly observed in males, with sex hormone regulation likely playing a pivotal role. Hcy has been shown to induce myocardial contractile dysfunction through mitochondrial calcium overload and ROS generation (36). Male cardiomyocytes, potentially attributable to higher metabolic demands fostered by androgens and a greater mitochondrial complement, may be more susceptible to Hcy-induced oxidative damage (11). Moreover, Hcy promotes fibroblast proliferation via the Akt/FoxO3 pathway, an effect that is synergistically amplified by androgens through enhanced TGF-β signaling. Additionally, estrogen is known to impact methionine/Hcy metabolism, with lower Hcy levels observed in pregnant, premenopausal, and postmenopausal women on estrogen replacement therapy (ERT) compared to age-matched men and postmenopausal women not on ERT (37). Estrogen may reduce Hcy levels and prevent its accumulation by interfering with the transsulfuration pathway, enhancing the net production of glutathione (GSH), and NO, thereby contributing to its beneficial effects on the vasculature (38).

Regarding cerebrovascular phenotypes, our findings revealed that women predominantly exhibited associations, including a negative correlation between genetically predicted Hcy levels and brain white matter volume. Previous studies have demonstrated that elevated Hcy levels are significantly associated with the severity of silent cerebral small vessel disease (SVD) and atherosclerotic/lipidic SVD in women, an association absent in men (39). Potential mechanisms for this sex-specific cerebrovascular vulnerability may relate to differences in vascular architecture and hormonal influences on the blood-brain barrier (BBB). Women have been reported to exhibit higher microvascular density in cortical gray matter and a smaller vessel caliber index (VSI) in the thalamic region compared to men (40). This relatively denser microvascular architecture may render the female cerebrovasculature more susceptible to Hcy-induced oxidative stress.

Our study demonstrated that elevated Hcy levels were robustly associated with increased risks of CVD, including vascular occlusion, subarachnoid hemorrhage, aortic aneurysm/dissection, atherosclerosis, cerebral infarction, IHD, diabetes, and related disorders. Epidemiological evidence also supported that HHcy as a systemic vascular risk amplifier, with significant associations identified across abdominal aortic aneurysm (41), stroke (42), coronary heart disease (43), and ischemic cerebrovascular events (44). Strikingly, the risks were pronounced only in individuals over 65 years, with no significant effects observed in younger populations. The age-specific exacerbation of HHcy-related risks may be primarily driven by three interrelated mechanisms: First, the dual phenomena of rising Hcy levels and amplified pathogenic sensitivity with advancing age exacerbates its detrimental vascular effects (32); Second, progressive vascular degeneration marked by endothelial dysfunction, oxidative stress, and arterial stiffening synergistically interacts with HHcy to potentiate structural deterioration. Thirdly, high prevalence of comorbidities such as hypertension, diabetes, chronic kidney disease in older adults, further exacerbates Hcy accumulation and perpetuating a cycle of metabolic and vascular dysfunction.

The present study evaluated the association between sulfur microbial diet and EAT-Lancet dietary patterns with genetically determined Hcy levels but revealed no significant correlations. This null finding may arise from complex interactions inherent to the dietary components and methodological limitations. First, while the EAT-Lancet diet is rich in folate (e.g., leafy greens), Hcy metabolism requires synergistic cofactors such as vitamin B6 and B12. Restriction of red meat, which is a primary source of vitamin B12 (45), might counteract the folate-mediated Hcy-lowering effects by inducing B12 deficiency, which is a known driver of HHcy. Similarly, the sulfur microbial diet, which may alter sulfur-containing amino acid metabolism, could paradoxically reduce Hcy precursors or modulate gut microbiota-dependent Hcy clearance pathways, potentially offsetting its theoretical benefits. Moreover, due to the unavailability of direct serum Hcy levels, we employed the wGRSs for Hcy as a proxy. While these genetic prediction of Hcy levels are correlated with actual measured levels, it is acknowledged that there are inherent limitations in fully representing the true levels given the influence of environmental factors (46). Additionally, the calculation of the wGRSs for Hcy levels was based on only 14 SNPs, and the limited number of SNPs and resultant small variance explained by these genetic instruments may have contributed to insufficient statistical power in our analysis (47). Third, recall bias in dietary assessments and the multifactorial regulation of Hcy levels likely attenuated the detectable impact of diet alone. Thus, while our data did not support strong dietary modulation of genetically predicted Hcy levels, the hypothesized pathways remain biologically plausible and warrant further investigation.

While our study focused on genetic and dietary modulation of Hcy, the broader context of food fortification policies warrants consideration. The United States mandated folate fortification initiated in 1998, subsequently adopted by Canada, Chile, Australia and other nations primarily to reduce neural tube defects (48). More recently, the UK government has announced plans to transition from voluntary to mandatory folic acid fortification (49). Population studies demonstrate that such policies reduce Hcy levels approximately by 50% (50) and contribute to clinical benefits including a decline in stroke mortality rates (51), and dementia prevention (52). longitudinal data from the Framingham Offspring Study cohort demonstrated significantly elevated mean serum folate concentrations among middle-aged and older adult participants (50). This finding aligns with our observation that Hcy-mediated structural damage exhibits predominant effects in older populations (>65 years), suggesting that folate fortification may be particularly crucial for attenuating age-associated vascular pathology in this vulnerable demographic. Our results provide compelling epidemiological evidence that could further inform ongoing policy discussions regarding mandatory folic acid fortification in the UK.

We further explored the relationship between two dietary patterns and CVD. Consistent with previous findings, the sulfur microbial diet exhibited a positive correlation with multiple cardiovascular and cerebrovascular pathologies (23, 24). However, the EAT-Lancet diet was associated with an increased risk of hypertension in individuals aged > 65 years, a finding that contrasts with an 18-year Chinese national cohort study reporting reduced hypertension risk with higher adherence to this diet (53). This discrepancy may stem from age-specific physiological vulnerabilities and the dietary composition of the EAT-Lancet framework. This diet emphasizes whole grains, vegetables, fruits, legumes, and nuts as primary energy sources while strictly limiting red meat ( ≤ 14 g/day) and processed meats (20), which aligns with its goal of promoting planetary and cardiovascular health. However, its restrictions on animal-derived foods, particularly red meat, dairy, and fish, may inadvertently compromise nutrient adequacy in older adults and lead to insufficient calcium, vitamin D, and ω-3 polyunsaturated fatty acids (PUFAs), impairing vascular elasticity and blood pressure regulation (54). Moreover, the limitation on animal protein may exacerbate age-related sarcopenia and frailty, as low protein intake is linked to muscle loss and metabolic dysfunction (55, 56). And strict red meat restriction may reduce taurine availability, which is a sulfur-containing amino acid critical for NO-mediated vasodilation and endothelial function.

This study presents the first comprehensive characterization of sex- and age-specific effects of genetically predicted Hcy levels on cardiocerebral structural alterations through integrative analysis of multi-omics datasets including genomic, imaging, and dietary profiles. Our findings systematically elucidate the triangular relationship among dietary factors, Hcy levels, and disease pathogenesis. The identification of differential Hcy-mediated mechanisms across demographic subgroups provides crucial evidence for developing precision clinical strategies. We specifically advocating for enhanced cardiac surveillance in male populations and prioritized cerebral small vessel disease screening in female cohorts. However, several limitations should be acknowledged. An important limitation of our study relates to the wGRS approach. While methodologically robust, our wGRS explained 3.22% of the variance in Hcy levels. This limited explanatory power reflects both the complex polygenic architecture of Hcy and inherent limitations of current GWAS-derived variants. The wGRS methodology may be subject to several potential biases, including incomplete linkage disequilibrium coverage of causal variants, possible pleiotropic effects of included SNPs, and population-specific differences in allele frequencies and effect sizes. Moreover, the reliance on genetically predicted Hcy levels as a proxy for actual measurements may introduce discrepancies, as genetic predictions may not perfectly align with measured Hcy concentrations.

Secondly, the intricate nature of dietary intake and the complex interplay of its metabolites within the human body may not be entirely represented by our derived diet scores. These score serve as a simplification of a multifaceted dietary pattern and may fail to account for all relevant sulfur-containing compounds or their metabolic pathways. Thirdly, the sulfur microbial diet was originally developed in a US male cohort and information on the microbiomes of the participants is not available in the UK Biobank, which prevent us from validating specific food-microbiome interactions. Additionally, the dietary measurements relied upon 24-h recall data, which are susceptible to recall bias and may not accurately reflect the participants' usual dietary habits. This suggests that future research should improved dietary assessment using repeated measures or real-time monitoring approaches to reduce measurement error and consider multi-omics intergration that combine genomics with metabolomics and microbiome data to better capture gene-diet interactions.

Our findings highlight the potential utility of cardiocerebral imaging traits as intermediate phenotypes bridging genetic and clinical risk. These structural metrics may serve as early biomarkers for risk stratification and guide personalized interventions in age- and sex-sensitive multimorbidity prevention.

## 5 Conclusion

In conclusion, our study provides robust evidence that genetically predicted Hcy levels exert sex- and age-specific effects on cardiocerebral structural phenotypes and age-specific effects on disease risks, with distinct cardiocerebral metrics acting as potential mediators in hypercholesterolemia and hypertension pathogenesis. Notably, while sulfur microbial and EAT-Lancet dietary patterns demonstrated direct associations with CVD, their effects were independent of Hcy modulation, highlighting the need to explore alternative pathways, such as gut microbiota-derived metabolites or systemic inflammation, in diet-disease relationships. As the first study to comprehensively characterize the multidimensional interactions between genetic Hcy determinants, cardiocerebral architecture, and dietary exposures, our work underscores critical clinical implications in precision prevention strategies and dietary guidelines.

## Data Availability

Publicly available datasets were analyzed in this study. The data analyzed in this study were obtained from the UK Biobank (https://www.ukbiobank.ac.uk) under Application Number 278107. Researchers can apply to use the UK Biobank dataset by registering and applying at: http://ukbiobank.ac.uk/register-apply/. Further inquiries can be directed to the corresponding authors.

## Supplementary material

Supplementary Figure 1 Mediation analysis of homocysteine-related brain/heart structural changes to cardiocerebrovascular and metabolic disorders among individuals aged < 65 years.

Supplementary Figure 2 Association between two diet patterns and risk of cardiocerebrovascular diseases, with sex-stratified analysis. **(A)** Association between sulfur microbial diet and risk of cardiocerebrovascular diseases, with sex-stratified analysis; **(B)** Association between EAT-Lancet diet and risk of cardiocerebrovascular diseases, with sex-stratified analysis.
