# Supplementary figures and images for: Genetic homocysteine risk shapes cardiocerebral structure and multimorbidity through age- and sex-specific mechanisms: a UK Biobank study
Source: Front Nutr. 2025 Nov 14;12:1637592. doi: 10.3389/fnut.2025.1637592 (PMC12661569; doi:10.3389/fnut.2025.1637592)

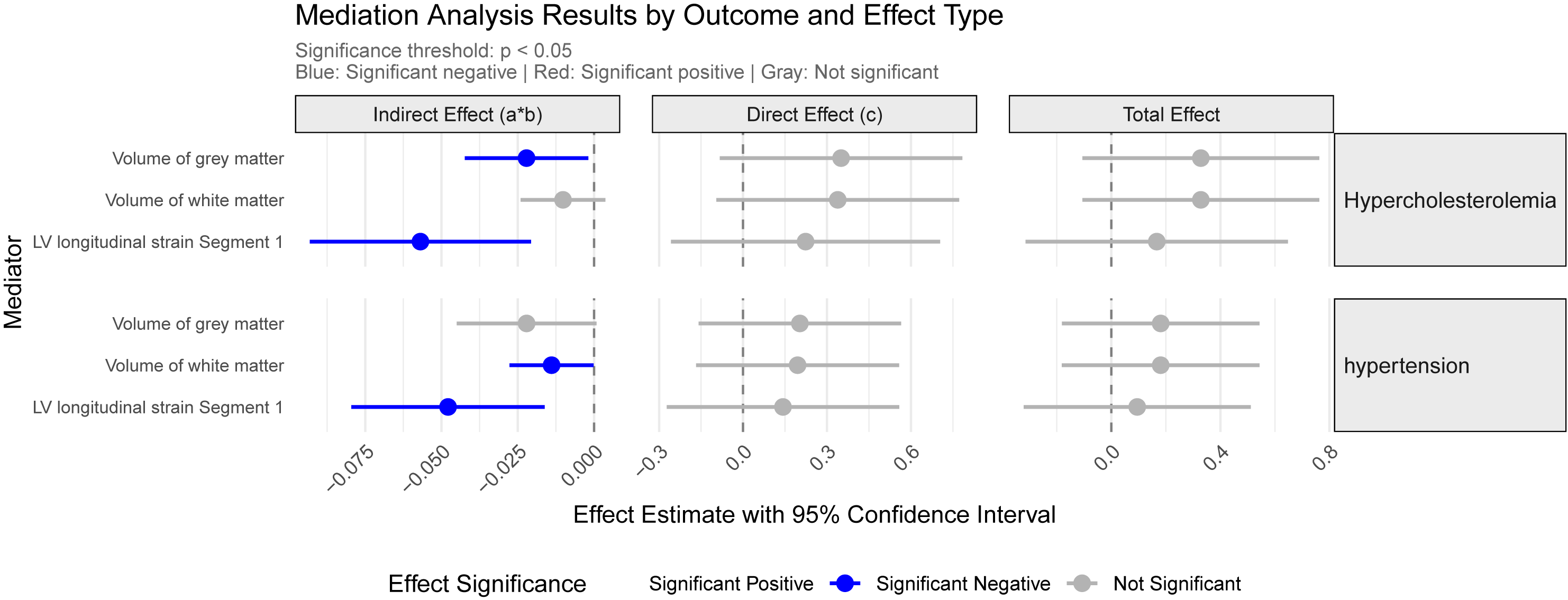

Supplement: Supplementary Figure 1 — Mediation analysis of homocysteine-related brain/heart structural changes to cardiocerebrovascular and metabolic disorders among individuals aged < 65 years. [file Image_1.tif]

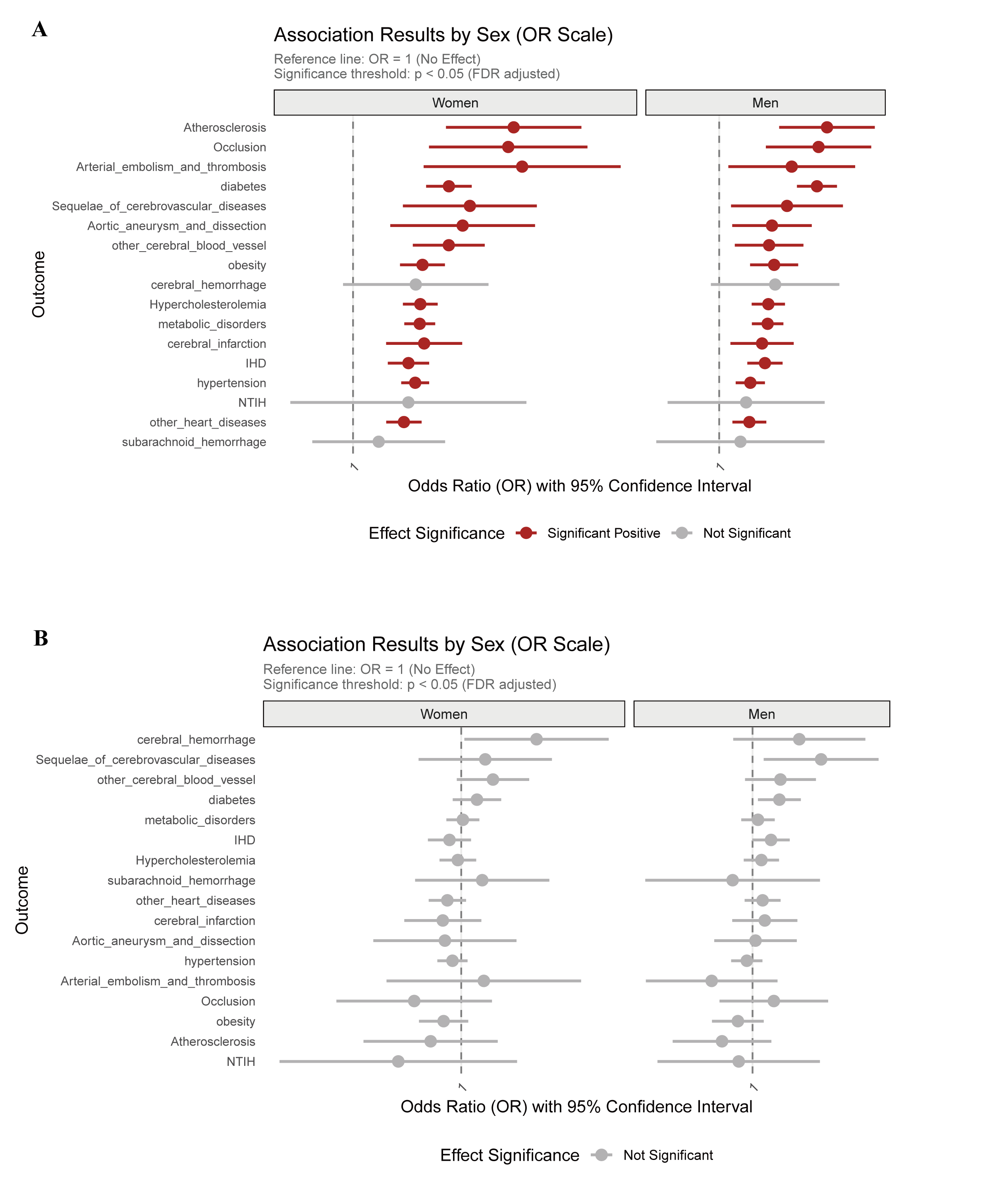

Supplement: Supplementary Figure 2 — Association between two diet patterns and risk of cardiocerebrovascular diseases, with sex-stratified analysis. (A) Association between sulfur microbial diet and risk of cardiocerebrovascular diseases, with sex-stratified analysis; (B) Association between EAT-Lancet diet and risk of cardiocerebrovascular diseases, with sex-stratified analysis. [file Image_2.tif]
